# Logistic regression analyses of factors affecting the euploidy of blastocysts undergoing in vitro fertilization and preimplantation genetic testing

**DOI:** 10.1097/MD.0000000000029774

**Published:** 2022-06-30

**Authors:** Zhiping Zhang, Lei Zhang, Yaoqin Wang, Xingyu Bi, Lixia Liang, Yuan Yuan, Dan Su, Xueqing Wu

**Affiliations:** a Center of Reproductive Medicine, Affiliated Children’s Hospital of Shanxi & Women Health Center of Shanxi Medicine University, Taiyuan, Shanxi, China.

**Keywords:** blastocyst trophectoderm biopsy, chromosomal abnormality, inversion, next-generation sequencing, preimplantation genetic testing, reciprocal translocation, Robertsonian translocation

## Abstract

Embryo chromosomal abnormalities are considered as the main cause of low pregnancy rate for in vitro fertilization (IVF). Recently, a new metric of success in assisted reproductive technology, that is, the ability to achieve at least 1 euploid blastocyst for transfer, has been brought into focus among clinicians. Our study aimed to investigate the effects of different factors on the euploidy of blastocysts undergoing IVF and preimplantation genetic testing (PGT).

This retrospective observational study included 493 cycles underwent IVF/intracytroplasmatic sperm injection intended to obtain trophectoderm biopsy for PGT from June 2016 to December 2019 at a single academic fertility center. Logistic regression was adopted to analyze the clinical characteristics and embryonic data related to the ability to achieve at least 1 euploid blastocyst for transfer.

The study took 1471 blastocysts from 493 cycles as samples for PGT. Among them, 149 cycles (30.22%) had no euploid blastocyst and 344 cycles (69.78%) had at least 1 euploid blastocyst. A multivariate logistic analysis suggested that maternal age >36, abnormal parental karyotype, nonfirst cycles and blastocysts number per cycle <3 were the risk factors for no euploid blastocyst.

The parental karyotype, maternal age, number of cycles, and number of blastocysts per cycle were the dominant factors affecting the ability to achieve at least 1 euploid blastocyst for transfer and therefore could be regarded as potential predictors for genetic counseling.

## 1. Introduction

The number of couples seeking help in assisted reproductive technologies (ART) is progressively increasing and about 1.5 million cycles are currently performed every year.^[1]^ Despite the notable developments in ART over the last decades, the live birth rates remain at about 40%. Moreover, the age of the population seeking ART is increasing steadily as both women and men are postponing child bearing. Aging couples, with high risk of abnormal chromosome of embryos,^[2]^ in turn, poses enormous challenges for clinicians and researchers.

Chromosome abnormalities are common in early human embryos such as the ones of day 3 to day 5. Embryo morphology score, which has currently been used as the common standard for embryo transfer, has, however, a weak correlation with embryonic chromosomes. Alfarawati et al^[3]^ found a significant proportion of aneuploid embryos are capable of achieving the highest morphologic scores, and some euploid embryos are of poor morphology. Pellicer’s research showed that a remarkable rate of embryos with chromosomal abnormalities was able to develop to the blastocyst with suitable morphology parameters; in fact, 42.8% of chromosomally abnormal embryos and 53.7% of mosaic embryos reached blastocyst stage.^[4]^ Embryo chromosomal abnormalities are considered to be the main cause of low pregnancy rate for in vitro fertilization (IVF) as it can directly lead to implantation of an abnormal conceptus, resulting in early miscarriage, late abortion, or the delivery of an affected child.^[5]^ So, raising the success rate of IVF treatments is still a challenge that requires a reliable mean to identify the embryos most potential for pregnancy.

Preimplantation genetic testing (PGT) helps patients to select embryos free of chromosome abnormalities and monogenic diseases. PGT has been applied for the past over 10 years to assess the chromosomal abnormalities of embryos to satisfy specific patient groups with reproductive outcomes. Accurate diagnosis can improve the success rates of implantation and live birth and then reduce the risk of miscarriage.

In this study, we used next-generation sequencing technology to screen the chromosome formation characteristics of blastocyst-stage embryos. By investigating the effects of the clinical characteristics and embryonic data on the euploidy of blastocysts undergoing IVF and PGT, the study would provide reliable basis for both patient counseling and clinicians to make a working plan with a clear management goal.

## 2. Materials and methods

### 2.1. Subjects and study design

Retrospective observational study included couples undergoing IVF and PGT cycles from June 2016 to December 2019 at a Center of Reproductive Medicine, Affiliated Children’s Hospital of Shanxi & Women Health Center of Shanxi Medicine University, Taiyuan, Shanxi, China. PGT-aneuploidy was used for 222 cases suffering advanced maternal age, severe male factor infertility, recurrent miscarriage, or repeated implantation failure. PGT-Structural rearrangement (PGT-SR) was used for 82 cases in which one partner of each couple carried a Robertsonian translocation, 174 cases with one partner carrying reciprocal translocation and 15 cases with one partner carrying inversion. Women who underwent PGT for single-gene diseases, women receiving PGT on day 3 embryos for biopsy, patients who had treatment involving oocyte donation or sperm donation and patients who had PGT on frozen-thawed blastocysts were excluded from the study.

Baseline characteristics included: female age, male age, parental genetic backgrounds, infertility duration, type of infertility, number of pregnancy loss, body mass indexes, and basal serum values follicle-stimulating hormone (FSH), luteinizing hormone, and E2. Treatment characteristics included: the type of controlled ovarian hyperstimulation regimen, total gonadotropin dose, and days of gonadotropin. Embryo laboratory indicators included: number of oocytes retrieved, number of mature (MII) oocytes retrieved, rate of MII oocytes, rate of 2-pronuclei zygotes, number of embryos per oocyte retrieval, number of high-quality embryos per oocyte retrieval, number of blastocysts, and number of euploid blastocysts (Table S1, Supplemental Digital Content, http://links.lww.com/MD/G810).

### 2.2. Blastocyst biopsy and whole genomic amplification

Five to 10 cells were taken from the trophectoderm on day 5 or 6 of embryos at the blastocyst stage. Whole genomic amplification was conducted by the multiple annealing and looping based amplification cycles method according to the manufacturer’s standard protocol (Yikon Genomics Inc, China). The biopsied cells were transferred carefully into 5 μL of the Lysis Reaction Mix in a polymerase chain reaction tube. Then the cell was lysed by heating and sequential amplification.

### 2.3. Chromosomal copy number variations by next-generation sequencing and validation

Chromosomal copy number variation (CNV) analyses were performed as previously described.^[6]^ Following purification of the products and library preparation, the amplified genome of each sample was sequenced, yielding at least ~2× sequencing depth and for a total of ~2 million raw reads and 5 million raw reads in PGT-aneuploidy and PGT-SR on Proton Life DA8600 platform with single-ended read length of 40 bp. Each embryo was subjected to a genome-wide CNVs analysis to determine the euploidy or aneuploidy status. Embryos with CNVs ≥4M will be identified as aneuploidy and abnormal chromosome segments ≥1M can be detected near the chromosome break point for PGT-SR.

### 2.4. Statistical analysis

Statistical analysis was performed using STATA software version 13.0 (Stata Corporation, College Station, TX). Differences between no euploid blastocysts group and at least 1 euploid blastocyst group were analyzed by the χ^2^ test or Fisher exact test as appropriate. Logistic regression analysis was applied to identify dominant factors. A *P* value <0.05 was reported as statistical significance.

### 2.5. Ethics approval

The Ethics Committee of Affiliated Children’s Hospital of Shanxi & Women Health Center of Shanxi Medicine University, China, approved the study [ID:IRB-KY-2020-013 (017)]. All of the women involved in the study were treated at this center and routinely provided with informed consents for their clinical data as anonymous record to be used for research purposes.

## 3. Result

The total samples included 1471 blastocysts from 493 cycles. Among them, 149 cycles (30.22%) had no euploid blastocyst and 344 cycles (69.78%) had at least 1 euploid blastocyst. The clinical characteristics and embryonic data of the 493 cycles are shown in Table 1 and Table 2, respectively.

**Table 1:** Clinical characteristics of 493 couples included in the study.

Characteristics	
All cycles, n	493
Maternal age, yr, mean (SD)	31.91 (4.80)
≤36, n (%)	406 (82.35)
>36, n (%)	87 (17.65)
Paternal age, yr, mean (SD)	33.41(5.57)
<40, n (%)	429 (87.02)
≥40, n (%)	64 (12.98)
Parental genetic backgrounds, n	493
Inversion, n (%)	15 (3.04)
Reciprocal translocation, n (%)	174 (35.29)
Robertsonian translocation, n (%)	82 (16.63)
Normal parental karyotype, n (%)	222 (45.03)
Duration of infertility, yr, median (range)	2 (0.3–16)
<2, n (%)	268 (54.36)
≥2, n (%)	225 (45.64)
Type of infertility	
Primary infertility, n (%)	144 (29.21)
Secondary infertility, n (%)	349 (70.79)
Number of pregnancy loss, median (range)	1 (0–6)
<2, n (%)	277 (56.19)
≥2, n (%)	216 (43.81)
Number of cycles	
1 (first cycle), n (%)	265 (53.75)
≥2 (nonfirst cycle), n (%)	228 (46.25)
BMI, kg/m^2^, n (mean, SD)	493 (23.09, 3.15)
≤18.5	21 (4.26)
18.5–23.99	302 (61.26)
24–27.99	138 (27.99)
>28	32 (6.49)
Basal serum FSH, IU/L, n (mean, SD)	493 (8.22, 2.78)
≤6.7	126 (25.56)
6.7–8.0	125 (25.35)
8.0–9.3	111 (22.52)
>9.3	131 (26.57)
Basal serum LH, IU/L, n (mean, SD)	493 (4.62, 2.78)
≤3.1	130 (26.37)
3.1–4.2	120 (24.34)
4.2–5.4	120 (24.34)
>5.4	123 (24.95)
Basal serum E2, pg/mL, n (mean, SD)	493 (72.13, 39.84)
≤50.20	124 (25.15)
50.21–64.8	123 (24.95)
64.81–82	124 (25.15)
>82	122 (24.75)
Type of COH regimen, n (%)	
Long-term protocol	219 (44.42)
Short-term protocol	126 (25.56)
Antagonist protocol	89 (18.05)
Others	59 (11.97)
Total Gn, median (range)	2850 (300–9075)
≤2400	147 (29.82)
2400–2850	108 (21.91)
2850–3525	119 (24.14)
>3525	119 (24.14)
Days of Gn, median (range)	10 (2–24)
≤9	168 (34.08)
10–11	161 (32.66)
≥12	164 (33.27)

BMI = body mass indexes, COH = controlled ovarian hyperstimulation, FSH = follicle-stimulating hormone, Gn = gonadotropin, LH = luteinizing hormone, SD = standard deviation.

**Table 2:** Embryo laboratory indicators of 493 couples included in the study.

Embryo laboratory indicators	
Number of retrieved oocytes, n (median, range)	493 (13, 1–64)
≤8	147 (29.82)
9–13	101 (20.49)
14–19	135 (27.38)
>19	110 (22.31)
Number of mature oocytes (MII) per oocyte retrieval, n (median, range)	493 (10, 1–48)
≤6	125 (25.35)
7–10	123 (24.95)
11–16	134 (27.18)
>16	111 (22.52)
Rate of MII oocytes, n (median, range)	493 (0.86, 0.25–1)
<0.86	247 (50.10)
≥0.86	246 (49.90)
Rate of two-pronuclei zygotes, n (median, range)	493 (0.80, 0–1)
<0.80	241 (48.88)
≥0.80	252 (51.12)
Embryos per oocyte retrieval, n (median, range)	493 (4, 0–23)
≤3	163 (33.06)
4–5	120 (24.34)
6–7	102 (20.69)
>7	108 (21.91)
High-quality embryos per oocyte retrieval, n (median, range)	493 (4, 0–23)
≤2	167 (33.87)
3–4	125 (25.35)
5–6	80 (16.23)
>6	121 (24.54)
Number of blastocysts per cycle, n (median, range)	493 (2, 0–13)
≤2	261 (52.94)
>2	232 (47.06)

Univariate analysis evaluated the probability of having at least 1 euploid blastocyst available for transfer (Table 3).Compared with the at least 1 blastocysts group, the no euploid blastocysts group had more maternal aged >36 (*P* < 0.001), more paternal aged ≥40 (*P* < 0.001), and less normal parental karyotype (36.24% vs 48.84%, *P*<0.05). The no euploid blastocysts group were more likely to have (1) less number of pregnancy loss, (2) a longer duration of infertility, (3) more nonfirst cycles, (4) higher basal serum FSH, (5) less classical long protocol of controlled ovarian hyperstimulation, and (6) less number of oocytes retrieved, less number of mature (MII) oocytes retrieved, less number of embryos per oocyte retrieval, less number of high-quality embryos per oocyte retrieval and less number of blastocysts than at least 1 euploid blastocysts group (*P* < 0.05). The other variables were not statistically significant (*P* > 0.05).

**Table 3:** Related factors affecting the euploidy blastocysts.

Variate	Category	No euploid blastocyst group, n (%)	At last 1 euploid blastocyst group, n (%)	χ^2^	*P* value
Maternal age, yr	≤36	100 (67.11)	306 (88.95)	34.12	0
	>36	49 (32.89)	60 (11.05)	–	–
Paternal age, yr	<40	110 (73.83)	319 (92.73)	32.9	0
	≥40	39 (26.17)	25 (7.27)	–	–
Parental genetic backgrounds	Inversion	2 (1.34)	13 (3.78)	10.27	0.016
	Reciprocal translocation	64 (42.95)	110 (31.98)	–	–
	Roche translocation	29 (19.46)	53 (15.41)	–	–
	Normal parental karyotype	54 (36.24)	168 (48.84)	–	–
Duration of infertility, yr	<2	61 (40.94)	207 (60.17)	15.5	0
	≥2	88 (59.06)	137 (39.83)	–	–
Type of infertility	Primary infertility	49 (32.89)	95 (27.62)	1.4	0.24
	Secondary infertility	100 (67.11)	249 (72.38)	–	–
Number of pregnancy loss, n	<2	94 (63.09)	183 (53.20)	4.13	0.042
	≥2	55 (36.91)	161 (46.80)	–	–
Number of cycles, n	1 (first cycle)	59 (39.60)	206 (59.88)	17.21	0
	≥2 (nonfirst cycle)	90 (60.40)	138 (40.12)	–	–
BMI, kg/m^2^	≤18.5	9 (6.04)	12 (3.49)	5.01	0.17
	18.5–23.99	81 (54.36)	221 (64.24)	–	–
	24–27.99	47 (31.54)	91 (26.45)	–	–
	>28	12 (8.05)	20 (5.81)	–	–
Basal serum FSH, IU/L	≤6.7	28 (18.79)	98 (28.49)	18.87	0
	6.7–8.0	40 (26.85)	85 (24.71)	–	–
	8.0–9.3	24 (16.11)	87 (25.29)	–	–
	>9.3	57 (38.26)	74 (21.51)	–	–
Basal serum LH, IU/L	≤3.1	41 (27.52)	89 (25.87)	4.62	0.202
	3.1–4.2	27 (18.12)	93 (27.03)	–	–
	4.2–5.4	40 (26.85)	80 (23.26)	–	–
	>5.4	41 (27.52)	82 (23.84)	–	–
Basal serum E2, pmol/L	≤50.20	42 (28.19)	82 (23.84)	2.98	0.39
	50.21–64.8	34 (22.82)	89 (25.87)	–	–
	64.81–82	32 (21.48)	92 (26.74)	–	–
	>82	41 (27.52)	81 (23.55)	–	–
Type of COH regimen	Long-term protocol	48 (32.21)	171 (49.71)	14.77	0.002
	Short-term protocol	49 (32.89)	77 (22.38)	–	–
	Antagonist protocol	28 (18.79)	61 (17.73)	–	–
	Others	24 (16.11)	35 (10.17)	–	–
Total Gn, IU	≤2400	48 (32.21)	99 (28.78)	4.85	0.205
	2400–2850	25 (16.78)	83 (24.13)	–	–
	2850–3525	34 (22.82)	85 (24.71)	–	–
	>3525	42 (28.19)	77 (22.38)	–	–
Days of Gn, d	≤9	52 (34.90)	116 (33.72)	0.61	0.74
	10–11	45 (30.20)	116 (33.72)	–	–
	≥12	52 (34.90)	112 (32.56)	–	–
Number of retrieved oocytes	≤8	79 (53.02)	68 (19.77)	63.07	0
	9–13	24 (16.11)	77 (22.38)	–	–
	14–19	35 (23.49)	100 (29.07)	–	–
	>19	11 (7.38)	99 (28.78)	–	–
Number of mature oocytes (MII) per oocyte retrieval	≤6	72 (48.32)	53 (15.41)	71.82	0
	7–10	39 (26.17)	84 (24.42)	–	–
	11–16	26 (17.45)	108 (31.40)	–	–
	>16	12 (8.05)	99 (28.78)	–	–
Rate of MII oocytes	<0.86	72 (48.32)	175 (50.87)	0.27	0.6
	≥0.86	77 (51.68)	169 (49.13)	–	–
Rate of 2-pronuclei zygotes	<0.80	75 (50.34)	166 (48.26)	0.18	0.67
	≥0.80	74 (49.66)	178 (51.74)	–	–
Embryos per oocyte retrieval	≤3	87 (58.39)	76 (22.09)	73.14	0
	4–5	36 (24.16)	84 (24.42)	–	–
	6–7	13 (8.72)	89 (25.87)	–	–
	>7	13 (8.72)	95 (27.62)	–	–
High-quality embryos per oocyte retrieval	≤2	79 (53.02)	88 (25.58)	49.16	0
	3–4	42 (28.19)	83 (24.13)	–	–
	5–6	12 (8.05)	68 (19.77)	–	–
	>6	16 (10.74)	105 (30.52)	–	–
Number of blastocysts per cycle	≤2	124 (83.22)	137 (39.83)	78.59	0
	>2	25 (16.78)	207 (60.17)	–	–

BMI = body mass indexes, COH = controlled ovarian hyperstimulation, FSH = follicle-stimulating hormone, Gn = gonadotropin, LH = luteinizing hormone.

A multivariate logistical regression analysis was carried out based on the meaningful variables (*P* < 0.05) of univariate logistical regression analysis. Logistic regression methods were used in selecting model variables (Table 4). Abnormal parental karyotype, maternal age >36 years, nonfirst cycle, number of blastocysts per cycle <3 were the risk factors for no euploid blastocyst in this study (*P* < 0.05), but other variables were excluded in the model, as they did not have a significant relationship with the euploidy of blastocysts.

**Table 4:** Logistic regression of the significant variables in the no euploid and at last 1 euploid blastocysts groups.

Variate	Category	Estimate	Standard error	χ^2^	*P* value	OR (95% CI)
Maternal age	≤37 yr	−1.35	0.464	8.541	0.003	0.259 (0.104 to 0.643)
	>36 yr	Reference	–	–	–	–
Paternal age	<40 yr	−0.378	0.456	0.685	0.408	0.685 (0.280 to 1.675)
	≥40 yr	Reference	–	–	–	–
Parental genetic backgrounds	Inversion	−0.933	1.007	36.164	0	0.393 (0.055 to 2.834)
	Reciprocal translocation	1.808	0.341	–	–	6.100 (3.124 to 11.913)
	Robertsonian translocation	0.781	0.394	–	–	2.183 (1.009 to 4.723)
	Normal parental karyotype	Reference	–	–	–	–
Duration of infertility, yr	<2	−0.272	0.311	0.771	0.38	0.762 (0.415 to 1.401)
	≥2	Reference	–	–	–	–
Number of pregnancy loss, n	<2	0.064	0.285	0.05	0.822	1.066 (0.610 to 1.862)
	≥2	Reference	–	–	–	–
Number of cycles, n	1 (first cycle)	−0.672	0.26	6.759	0.009	0.511 (0.307 to 0.850)
	≥2 (nonfirst cycle)	Reference	–	–	–	–
Basal serum FSH, IU/L	≤6.7	−0.531	0.37	3.258	0.354	0.588 (0.285 to 1.214)
	6.7–8.0	0.031	0.358	–	–	1.031 (0.511 to 2.080)
	8.0–9.3	−0.317	0.374	–	–	0.728 (0.350 to 1.514)
	>9.3	Reference	–	–	–	–
Protocal of COH	Long	−0.548	0.394	3.216	0.36	0.578 (0.267 to 1.252)
	Short	−0.073	0.412	–	–	0.930 (0.415 to 2.084)
	Antagonist	−0.129	0.438	–	–	0.879 (0.373 to 2.072)
	Others	Reference	–	–	–	–
Number of retrieved oocytes, n	≤8	−0.38	0.817	6.258	0.1	0.684 (0.138 to 3.393)
	9–13	−0.362	0.717	–	–	0.697 (0.171 to 2.840)
	14–19	0.69	0.58	–	–	1.994 (0.639 to 6.218)
	>19	Reference	–	–	–	–
Number of mature oocytes per oocyte retrieval, n	≤6	1.43	0.886	4.622	0.202	0.107 (4.179 to 0.736)
	7–10	0.831	0.734	–	–	2.296 (0.545 to 9.673)
	11–16	0.007	0.602	–	–	1.007 (0.310 to 3.274)
	>16	Reference	–	–	–	–
Embryos per oocyte retrieval	≤3	0.887	0.933	1.492	0.684	2.427 (0.390 to 15.121)
	4–5	0.459	0.845	–	–	1.583 (0.302 to 8.295)
	6–7	0.117	0.615	–	–	1.124 (0.337 to 3.749)
	>7	Reference	–	–	–	–
High-quality embryos per oocyte retrieval	≤2	–0.504	0.394	1.283	0.733	0.578 (0.267 to 1.252)
	3–4	–0.556	0.412	–	–	0.930 (0.415 to 2.084)
	5–6	–0.687	0.438	–	–	0.879 (0.373 to 2.072)
	>6	Reference	–	–	–	–
Number of blastocysts	≤2	1.502	0.34	21.052	0.003	4.488 (2.307 to 8.735)
	>2	Reference	–	–	–	–

CI = confidence interval, COH = controlled ovarian hyperstimulation, FSH = follicle-stimulating hormone, OR = odds ratio.

The odds ratio for female age (≤36 vs >36) was 0.259 (95% confidence interval [CI], 0.104–0.643), whereas for number of cycles (1 vs ≥2) and number of blastocysts per cycle (≤2 vs >2) it was 0.511 (95% CI, 0.307–0.850) and 4.488 (95% CI, 2.307–8.735), respectively (*P* < 0.05). With regard to parental genetic backgrounds, the odds ratios for inversion versus normal parental karyotype, reciprocal translocation versus normal parental karyotype, and Robertsonian translocation versus normal parental karyotype were 0.393 (95% CI, 0.055–2.834), 6.100 (95% CI, 3.124–11.913), and 2.183 (95% CI, 1.009–4.723), respectively (*P*<0.05). This indicates that for 2 similar aged women at similar age, normal parental karyotype, first cycle, and more blastocysts per cycle would lead to increased probability of having at least 1 euploid blastocyst for the embryo transfer.

## 4. Discussion

The live birth rate has traditionally been used as an indicator of the success of ART treatment. However, with the improvement in blastocyst culture and embryo cryopreservation technologies and the widespread use of PGT, the evaluation of embryos with genetic normality is becoming a new effective alternative. In 2016, for example, the patient-oriented strategies encompassing individualized oocyte number collaborative group proposed a new metric of success in ART, namely, the ability to obtain the number of oocytes needed to achieve at least 1 euploid blastocyst for transfer.^[7]^ As a result, euploid blastocyst transplantation is increasingly popular among clinicians. Given that the transplantation of genetically normal embryos is not only the most important criterion for ART but also a key to success in IVF, it is essential to clarify the occurrence of euploid embryo for genetic counseling.

Genetic abnormalities in embryos are common and related to many factors. Women with advanced maternal age, experiencing recurrent spontaneous abortion or repeated implantation failure confront a high risk of developing embryos of chromosomal abnormalities. Besides, individuals carrying abnormal chromosome such as translocations or inversions are at high risk of abnormal gametes and thereby suffer reduced fertility or soaring spontaneous abortions.^[8,9]^ Globally, PGT is offered for women with advanced maternal age, having a history of recurrent abortions and implantation failures and couples with inherited Robertsonian translocations, reciprocal translocations or inversions to achieve a healthy live birth.

It is well known that there is an association between advanced maternal age and increased risk of chromosomal abnormalities in embryos, and many studies have proved that. Minasi et al^[10]^ found that the aneuploidy rate rises by 10% per female age group, with 48.1%, 41.3%, 29.7%, and 10.3% euploid blastocysts in patients with mean female ages ≤32, 33 to 36, 37 to 41, and ≥42, respectively. Franasiak et al^[11]^ performed a study on 15,169 consecutive trophectoderm biopsies from 2701 female patients aged 22 to 49. It was found that the rate of aneuploidy rises steadily with age, with the lowest risk at age of 26 to 30, and older women have an increased risk of aneuploidy. Indeed, transfer of euploid embryos markedly reduces the age-related drop in implantation rates.^[12–14]^ This study showed that maternal age above 36 is a risk factor for no euploid blastocysts with a *P* value <0.05 in both univariate and multivariate logistic regression models.

Advanced maternal age is often accompanied by a decline in ovarian function. Some evidence in literatures shows that, the quantity and quality of ovarian reserve play a role in embryonic euploidy. Women with aneuploid spontaneous miscarriage had a reduced ovarian reserve compared with those with euploid miscarriage.^[15]^ Similarly, mothers of children with trisomy 21 have significantly higher serum levels of FSH (indicating low ovarian reserve) than age-matched control subjects.^[16,17]^ La Marca et al^[18]^ found that female age and AMH are independently associated with the rate of euploid blastocysts. Our research showed that basal serum FSH affected the euploidy of blastocysts in univariate analysis but was not statistically significant in multivariate logistic regression models. The basal serum FSH might not have a direct effect on aneuploidy, nevertheless, it was associated with maternal age, which, in turn is associated with blastocyst aneuploidy.

Recurrent miscarriage is also a common indication for PGT. Dai et al^[19]^ investigated the fetal chromosomal copy number variations of 434 women with a history of 1 SA and 776 women with a history of more than 1 SA, they found there were no significant differences in the rates of chromosomal abnormalities according to the abortion frequency (*P* > 0.05). In this study, we found an opposite conclusion that the less number of pregnancy loss (<2 vs ≥2), the more risk of no euploid blastocyst for transfer in univariate analysis. We analyzed the subjects included in the 2 studies. Dai et al^[19]^ sampled naturally conceived fetus with spontaneous abortion, while we sampled patients with years-long infertility symptoms requiring ART. Of our patients, 51.93% couples (256/493) showed that one parent carried reciprocal translocation or Roche translocation, which were at a higher risk of no euploid embryos for transfer. There is 62.89% (161/256) couples had none or only once pregnancy loss. Therefore, the high risk of no euploid blastocyst for transfer may arise from parental chromosomal abnormalities rather than fewer abortions. The number of pregnancy loss was not an independent factor determining the euploidy of blastocysts when all covariates were included in the multivariate logistic regression models. This is consistent with Dai’s study.

Repeated implantation failure usually requires PGT to assist pregnancy. So, does the number of cycles affect the rate of euploid blastocyst? To our knowledge, there is little research concerning the impact of the number of cycles on the euploidy of blastocysts. In this study, no euploid blastocyst group had more nonfirst cycle than at least 1 euploid blastocyst group (*P*<0.05). And the odds ratio for number of cycles (first cycle vs nonfirst cycle) was 0.511 (95% CI, 0.307–0.850). Therefore, nonfirst cycle had a higher risk of having no transplantable blastocyst than first cycle.

The offspring of balanced translocation carrier, such as reciprocal translocations and Robertsonian translocation, have an increased risk of chromosomal abnormalities given the high occurrence of unbalanced gametes. Several studies have reported the prevalence of normal or balanced embryos in reciprocal translocations patients undergoing PGT ranges from 12.5% to 45 % in the cleavage stage and 28% to 53% in the blastocyst stage; and that in Robertsonian translocation patients undergoing PGT ranges from 38.7% to 55.1% in the cleavage stage and 50% to 74.1% in the blastocyst stage.^[20–25]^ Young et al^[26]^ found carriers of balanced chromosome inversions did not exhibit higher aneuploidy rates for chromosomes that were not involved in the inversion compared to maternal age-matched controls, signifying the absence of an interchromosomal effect. Our research shows that the odds ratio for parental genetic backgrounds (inversion vs normal parental karyotype, reciprocal translocation vs normal parental karyotype, Robertsonian translocation vs normal parental karyotype) was 0.393 (95% CI, 0.055–2.834), 6.100 (95% CI, 3.124–11.913), and 2.183 (95% CI, 1.009–4.723), respectively (*P* < 0.05). Thus, compared with normal parental karyotypes, the offspring of the Robertsonian translocation and reciprocal translocations had an increased risk of aneuploidy, while the offspring of the inversion had no such increased risk. This is consistent with a previous study.^[26]^ However, due to the small sample size of the inversion group included in this study, a more exact relationship between inversion carriers and the euploidy of blastocysts needs to be confirmed by a larger sample size.

A previous report found that for any given probability of blastocyst euploidy, the higher the number of MII oocytes, the higher the chances of having at least 1 euploid blastocyst within the patient embryo cohort.^[27]^ In this study, several embryo laboratory indicators including number of retrieved oocytes, number of mature oocytes (MII) per oocyte retrieval, embryos per oocyte retrieval, high-quality embryos per oocyte retrieval and number of blastocysts per cycle had a significant relationship with the euploidy blastocysts in univariate logistic regression models, but when all covariates were included in the bivariate logistic regression models, only the number of blastocysts was a significant risk factor for no euploid blastocysts. As is known, the number of mature oocytes positively affected the total number of euploid blastocysts per patient.^[18]^ The number of mature oocytes was positively correlated with the total number of euploid blastocysts, which, however, did not fully represent that the rate of having at least 1 euploid blastocyst would change with an increase in number of mature oocytes. A possible explanation is that the developmental potential of oocytes and embryos is more important for obtaining the sufficient MII oocytes. Maybe it is need to form a sufficient number of blastocysts to obtain at least one euploid embryo. Therefore, clinicians ought to adjust the controlled ovarian hyperstimulation regimen according to the patient’s situation and pursue quality rather than quantity of oocytes.

In this study, the type of controlled ovarian hyperstimulation regimen affected the euploidy of blastocysts in univariate analysis but was not statistically significant in multivariate logistic regression models. Long-term protocol was easier to obtain euploid embryos than the other protocols. It was because that the long-term protocol is being used more in patients with a good ovarian reserve function from the clinician’s perspective.. Therefore, clinicians do not necessarily prefer a specific regimen over others for obtaining more euploid embryos, while a regimen appropriate for patients is the best option.

Our predictive model is intended to serve both as a useful clinical tool for counseling infertile couples and a guide for clinicians to treat the patient with an optimal mindset to achieve euploid blastocysts.

## 5. Conclusion

In this study, the authors used a univariate logistic regression analysis to identify variables which are related with the probability of having at least 1 euploid blastocyst available for transfer. Then, these variables were included in the multivariate analysis, which showed that parental karyotype background, female age, number of cycles, and number of genetically analyzed blastocysts per cycle were independently associated with the euploidy of blastocysts. The high aneuploidy rates observed suggest that chromosome screening at the blastocyst stage may be beneficial, particularly in the case of advanced age females, nonfirst cycles, and couples carrying chromosomal abnormalities.

## Author contributions

Conceptualization: Xueqing Wu.

Data curation: Lei Zhang.

Investigation: Lixia Liang, Yuan Yuan, Dan Su, Zhiping Zhang.

Methodology: Lei Zhang.

Software: Zhiping Zhang, Yaoqin Wang.

Writing—original draft: Zhiping Zhang.

Writing—review and editing: Xingyu Bi, Xueqing Wu.

## Supplementary Material


